# Tumor-associated epilepsy in patients with brain metastases: necrosis-to-tumor ratio forecasts postoperative seizure freedom

**DOI:** 10.1007/s10143-021-01560-y

**Published:** 2021-05-14

**Authors:** Majd Bahna, Muriel Heimann, Christian Bode, Valeri Borger, Lars Eichhorn, Erdem Güresir, Motaz Hamed, Ulrich Herrlinger, Yon-Dschun Ko, Felix Lehmann, Anna-Laura Potthoff, Alexander Radbruch, Christina Schaub, Rainer Surges, Johannes Weller, Hartmut Vatter, Niklas Schäfer, Matthias Schneider, Patrick Schuss

**Affiliations:** 1 Department of Neurosurgery, Center of Integrated Oncology (CIO) Bonn, University Hospital Bonn, Venusberg-Campus 1, 53127 Bonn, Germany; 2 Department of Anesthesiology and Intensive Care Medicine, University Hospital Bonn, Bonn, Germany; 3 Department of Oncology and Hematology, Center of Integrated Oncology (CIO) Bonn, Johanniter Hospital Bonn, Bonn, Germany; 4 Department of Neuroradiology, University Hospital Bonn, Bonn, Germany; 5 Division of Clinical Neuro-Oncology, Department of Neurology, Center of Integrated Oncology (CIO) Bonn, University Hospital Bonn, Bonn, Germany; 6 Department of Epileptology, University Hospital Bonn, Bonn, Germany

**Keywords:** Epilepsy, Brain metastases, Cancer, Seizure freedom, ILAE

## Abstract

Surgical resection is highly effective in the treatment of tumor-related epilepsy (TRE) in patients with brain metastases (BM). Nevertheless, some patients suffer from postoperative persistent epilepsy which negatively impacts health-related quality of life. Therefore, early identification of patients with potentially unfavorable seizure outcome after BM resection is important. Patients with TRE that had undergone surgery for BM at the authors’ institution between 2013 and 2018 were analyzed with regard to preoperatively identifiable risk factors for unfavorable seizure outcome. Tumor tissue and tumor necrosis ratios were assessed volumetrically. According to the classification of the International League Against Epilepsy (ILAE), seizure outcome was categorized as favorable (ILAE 1) and unfavorable (ILAE 2–6) after 3 months in order to avoid potential interference with adjuvant cancer treatment. Among all 38 patients undergoing neurosurgical treatment for BM with concomitant TRE, 34 patients achieved a favorable seizure outcome (90%). Unfavorable seizure outcome was significantly associated with larger tumor volumes (*p* = 0.012), a midline shift > 7 mm (*p* = 0.025), and a necrosis/tumor volume ratio > 0.2 (*p* = 0.047). The present study identifies preoperatively collectable risk factors for unfavorable seizure outcome in patients with BM and TRE. This might enable to preselect for highly vulnerable patients with postoperative persistent epilepsy who might benefit from accompanying neuro-oncological expertise during further systemical treatment regimes.

**Supplementary Information:**

The online version contains supplementary material available at 10.1007/s10143-021-01560-y.

## Introduction

Seizures are among the most common symptoms in patients with brain tumors with a considerable incidence in patients with brain metastases (BM) albeit lower when compared to primary brain tumors [5]. This lower incidence seems to be related to the less infiltrative growth pattern resulting in more circumscribed lesions [22]. Neurosurgical treatment of BM not only reduces the intracranial tumor burden, but also provides excellent seizure control [34].

Nevertheless, some patients with surgically treated BM and tumor-related epilepsy (TRE) with unfavorable seizure outcome embark on further adjuvant therapy, which may itself have an epileptogenic effect [22]. Yet, recurrent seizures have a negative effect on the health-related quality of life (QoL), especially if they are not controlled postoperatively or with long-term medication only [14]. In common treatment settings, patients with primary cancer and newly diagnosed BM are referred back to the primary physician after successful neurosurgical management implementation of systemic treatment of the underlying malignancy. Since these patients with uncontrolled seizures despite surgery might benefit from continuous supportive neuro-oncological expertise accompanying their systemic therapy, their early identification is of crucial importance.

We have therefore reviewed our institutional database for possible preoperatively identifiable risk factors for unfavorable postoperative seizure outcome in patients with newly diagnosed BM and TRE.

## Materials and methods

Eligible for study inclusion were all patients (aged 18 years or older) with histopathologically proven brain metastasis requiring surgery and preoperative TRE who underwent neurosurgical resection of BM at the authors’ neuro-oncology specialty center between 2013 and 2018 and a sufficiently documented seizure history for at least 3 months postoperatively. Exclusion criteria, in addition to the absence of any of the above-mentioned requirements, were if the affected patients had a history of known epilepsy or if the seizures were attributable to another (known) cause. Following the ILAE definition, reported epilepsy was defined as (1) at least 2 unprovoked seizures occurring > 24 h apart, or (2) 1 unprovoked seizure and an increased probability of further seizures similar to the general risk of recurrence after 2 unprovoked seizures occurring in the next 10 years [7]. Therefore, patients with BM with both one and/or more than one symptomatic seizure were included in further analysis. Tumor-related epilepsy (TRE) was defined as newly recorded symptomatic seizures that occurred for the first time were associated with an MRI-based brain metastasis diagnosis, and the affected patient reported no history of previous epilepsy [15, 27]. Seizure semiology due to TRE is characterized as simple focal, complex focal, or generalized seizures [3, 8, 31]. Postoperative seizure outcome was evaluated for a period of up to 3 months after surgery to avoid possible interference with a postoperative adjuvant cancer treatment. Pertinent clinical information was collected and entered into a computerized database (SPSS, Version 25, IBM Corp., Armonk, NY). Information recorded included age, sex, localization and size of BM, information on multiple BM, seizure status and semiology (focal versus generalized) according to the criteria of the International League Against Epilepsy (ILAE) [32], underlying malignancy, and postoperative seizure outcome. The Karnofsky Performance Scale (KPS) was used to estimate a patient’s functional status in terms of daily living activity as well as to assess a replicable impression of the neurological impact of the tumor lesion preoperatively.

The indication for surgical treatment of a newly diagnosed intracranial metastasis is given at the weekly tumor board meeting, as previously reported [25].

Postoperative seizure control in terms of ILAE class I (completely seizure free, no auras) was considered as favorable seizure outcome. An unfavorable outcome was defined as ILAE class II–VI as previously described [24].

Routine imaging resection control was not performed in the absence of postoperative new neurologic deficit or evidence of intraoperative complications [2].

**Fig. 1:**
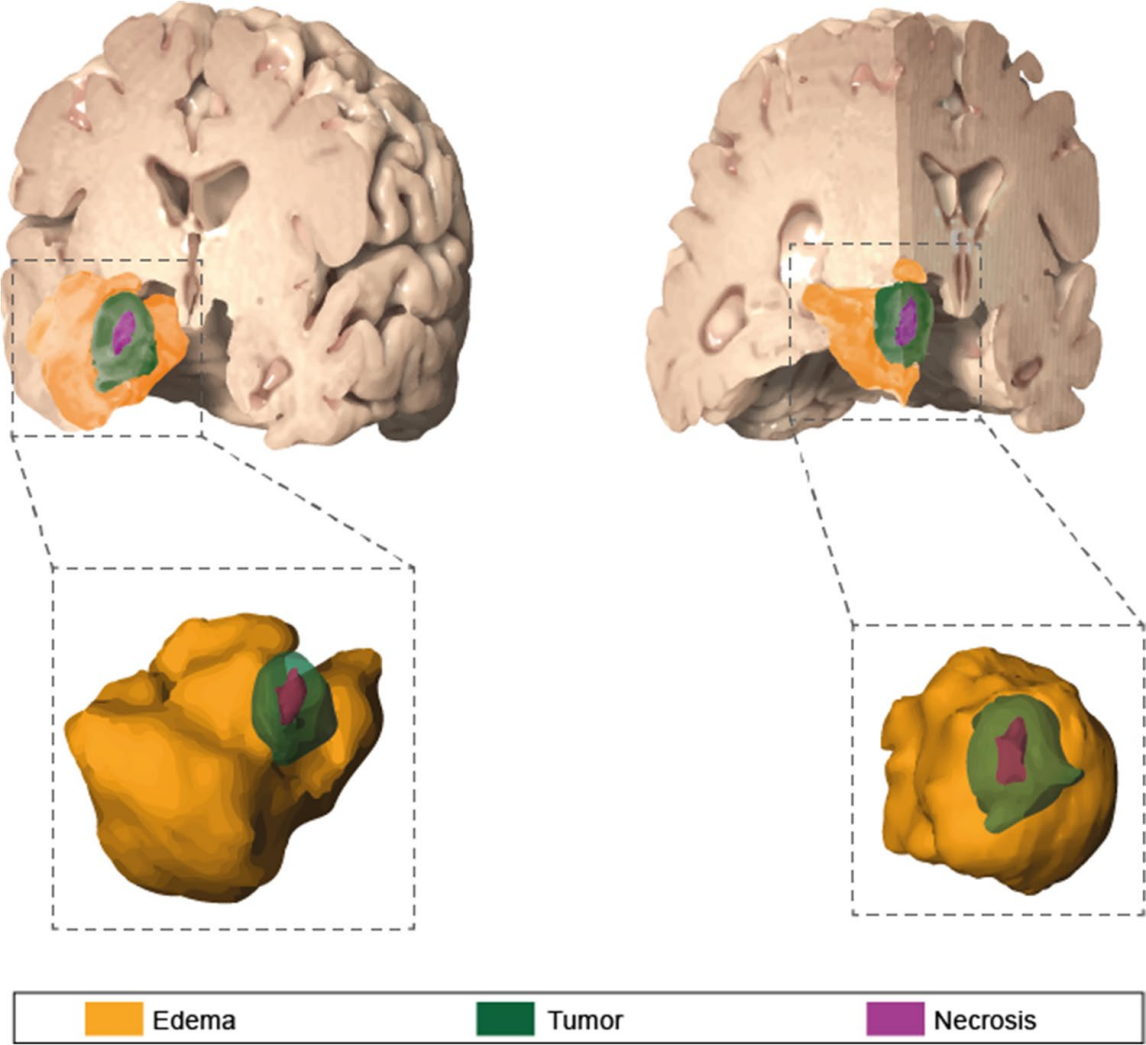
Illustration of metastatic tumor-, necrosis-, and tumor-related edema-volumes; right: frontal view; left: frontotemporal view

Volumetric analyses of preoperative contrast-enhancing tumor tissue, tumor necrosis, and perilesional brain edema were performed manually with commercially available software (TumorTracking Tool, IntelliSpace Portal 5.0, Philips, Best, Netherlands) by two authors (MB, MS). Any discrepancies were resolved in a consensus meeting with the senior author (PS). Therefore, enhancing volume on post-contrast T1 including central necrosis was considered as tumor. A non-enhancing region within the tumor on post-contrast T1 was determined as necrosis. The volume on T2/FLAIR hyperintensities surrounding the lesion, excluding the tumor volume, was classified as perilesional edema (Fig. 1). Two ratios were then calculated to better depict distinct aspects of each BM compartment: (1) necrosis/tumor-ratio (NTR; necrosis divided by tumor volume) and (2) edema/tumor-ratio (ETR; edema divided by tumor volume), as previously described by Henker et al. [10] (Fig. 1).

The study was approved by the local ethics committee. Because this study does not imply any burden for the patients and all data were retrieved from existing databases and registries, no informed consent had to be sought.

### Statistical analysis

Data analyses were performed using the SPSS computer software package (version 25, IBM Corp., Armonk, NY). Categorical variables were analyzed in contingency tables using Fisher’s exact test. The Mann–Whitney U-test was chosen to compare continuous variables as data were mostly not normally distributed. The area under the curve (AUC), as well as specificity and sensitivity, was determined using receiver operating characteristic (ROC) curves to identify a cut-off value of NTR for prediction of postoperative seizure outcome in the current patient population. Results with *p* < 0.05 were considered statistically significant.

## Results

### Patient characteristics and seizure outcome

We identified 38 patients with preoperative TRE who had undergone surgical treatment of BM at the authors’ institution between 2013 and 2018. In all patients, complete resection of the mass was performed, including two patients (5%) in whom a supramarginal resection regimen was followed through an anterior temporal lobe resection. Overall, 34 patients (90%) achieved favorable postoperative seizure outcome. Patients with unfavorable seizure outcome more frequently had generalized seizures and a shorter OS. However, differences between patients with favorable and unfavorable seizure outcome did not reach statistical significance (Table 1). Further details on histopathological findings, presence of multiple BM, seizure semiology, and antiepileptic drug regimen are provided in Supplementary Table S1.

**Table 1:** Patient characteristics

	Favorable seizure outcome (*n* = 34)	Unfavorable seizure outcome (*n* = 4)	
Median age at surgery (yrs)	61	54	*p* = 0.37
Female gender	19 (56%)	2 (50%)	*p* = 1.0
Preoperative KPS ≥ 70	29 (85%)	3 (75%)	*p* = 0.51
Preoperative seizure semiology			*p* = 0.29
Partial	21 (62%)	1 (25%)	
Generalized	13 (38%)	3 (75%)	
Primary site of cancer			
Lung	13 (38%)	3 (75%)	*p* = 0.29
Breast	5 (15%)	0 (0%)	*p* = 1.0
Melanoma	4 (12%)	0 (0%)	*p* = 1.0
Others	12 (35%)	1 (25%)	*p* = 1.0
Median OS (mo)	16 (95% CI 2.0–30.0)	8 (95% CI 2.1–13.9)	*p* = 0.28

*yrs*, years; *KPS*, Karnofsky Performance Scale; *OS*, overall survival; *mo*, months

### Analysis of imaging characteristics and postoperative seizure outcome

Temporal localization of surgically treated BM or hemorrhagic transformation did not significantly affect postoperative seizure outcome (*p* = 1.0) (Table 2). While multiple BM at the time of surgical treatment were more frequent among patients with an unfavorable seizure outcome, this difference did not reach statistical significance (*p* = 0.19). Of those patients who achieved a favorable seizure outcome, 6 patients (18%) already manifested multiple BM at initial presentation, whereas this was the case in 2 patients (50%) with unfavorable seizure outcome during follow-up (*p* = 0.2).

**Table 2:** Imaging-based analysis for factors influencing seizure outcome

	Favorable seizure outcome (*n* = 34)	Unfavorable seizure outcome (*n* = 4)	
Temporal location of BM	6 (18%)	1 (25%)	*p* = 1.0
Multiple BM	6 (18%)	2 (50%)	*p* = 0.19
Hemorrhagic transformation	8 (24%)	1 (25%)	*p* = 1.0
Preoperative midline shift ≥ 7 mm	1 (3%)	2 (50%)	*p* = 0.025, OR 33, 95% CI 2.0–538.7
Median tumor volume (cc)	0.2	3.1	*p* = 0.002
NTR > 0.2	2 (6%)	2 (50%)	*p* = 0.047, OR 16, 95% CI 1.4–180.9

*BM*, brain metastasis; *cc*, cm^3^; *NTR*, necrosis/tumor-ratio

Patients with an unfavorable seizure outcome presented with a significantly larger tumor volume compared to patients with favorable seizure outcome (median volume: 3.1 cc versus 0.2 cc; *p* = 0.002). The ROC analysis yielded a tumor volume cut-off of 1.7 cc regarding the correlation with a persisting postoperative seizure status (AUC = 0.98, SE = 0.02, *p* < 0.001, sensitivity 99%, specificity 80%). Furthermore, an initial midline shift of ≥ 7 mm showed a significant association with unfavorable postoperative seizure outcome compared to patients with a preoperative midline shift of < 7 mm (50% vs. 3%; *p* = 0.025) (Table 2).

The ROC analysis revealed a NTR cut-off value of 0.2 regarding the predictability of postoperative seizure-freedom (AUC = 0.81, SE = 0.14, *p* = 0.046; sensitivity 75%, specificity 65%). Subsequently, a NTR of > 0.2 was found to be significantly associated with unfavorable postoperative seizure outcome in further volumetric analysis (*p* = 0.047, OR 16, 95% CI 1.4–180.9) (Table 2).

## Discussion

Current evidence estimates that in about 4% of patients with epilepsy, the cause is the presence of a brain tumor. In contrast, the incidence of epilepsy in patients with brain tumor is about 30% [13]. However, seizure prevalence in brain tumors follows an inverse relationship related to tumor growth rate and associated malignancy [12]. Nevertheless, tumor-related epilepsy (whether due to primary brain tumor or BM) can massively reduce HRQoL in affected patients [13, 22]. Although the pathomechanisms of TRE are ultimately not yet conclusively established, several studies allude to changed neurotransmitter homeostasis in the tumor-surrounding brain area as a cause of TRE [35, 36]. In the various studies on alterations in glutamate or GABA transmissions within the peritumoral parenchyma, the focus is often on brain tumors and/or meningiomas [4, 17, 28]. Focusing on intracranial extraaxial lesions, such as brain metastases in this case, does not simplify the determination of the underlying pathomechanisms of TRE [11]. Nevertheless, brain metastases (as well as glioblastoma) are among the most rapidly growing intracranial lesions, so that here, in addition to various neurotransmitter alterations, sudden modes of tissue damage, such as both necrosis of cells and deposition of hemosiderin, are discussed as an underlying mechanism for TRE [21, 30]. Seizure semiology in patients with TRE is often related to tumor location. In general, partial seizures with tonic–clonic manifestation (with or without alteration of consciousness) are more common in patients with TRE [9, 16]. We found that neurosurgical treatment of BM could achieve postoperative seizure freedom in the majority of patients. This finding is in line with previous work [9, 22]. With regard to low- and/or high-grade gliomas, gross-total or supramarginal resection is also assuming an increasing role in the debate with regard to seizure outcome [19, 26]. In the case of BM, efforts are also accumulating to achieve the most complete resection results possible, even in more eloquent areas [20]. Although radiotherapy is another highly efficient therapeutic option in the case of BM and the incidence of TRE is lower, especially compared to low-grade gliomas, a future deepening of scientific efforts regarding the resection regimen also in BM would nevertheless be desirable. Patients with BM due to underlying melanoma or lung cancer exhibit the most frequent incidence of TRE among patients with BM [22, 29]. In the present study, no association was apparent between underlying cancer type and the preferential development of preoperative epilepsy in patients with BM. This may be due to patient selection, as the present data focused solely on patients with BM requiring surgery. Regarding antiepileptic drug (AED) regimens, further management with AED is often a matter of in-house philosophy given the sparse evidence in BM patients with TRE [20]. In line with the basic evidence-based recommendation regarding antiepileptic drugs, patients with TRE are often treated initially with monotherapy using levetiracetam or valproic acid (as was the case in this study), because the use of CYP3A4 coenzyme AEDs (such as carbamazepine, phenytoin, and phenobarbital) may interfere with the effectiveness of the necessary chemotherapy after surgery [7, 12, 18]. Due to the fact that patients with TRE often face a reduction in quality of life, stigmatization problems, and significant disabilities, close neurological/neuro-oncological engagement is desirable in BM patients for optimal management of TRE [15]. Likewise, for a better or more extensive research of the impact of tumor-associated epilepsy, including long-term medication if necessary, future research efforts should include more sophisticated testing methods (such as the Wechsler Adult Intelligence Scale) in the assessments of these patients. Furthermore, the present study highlights preoperative imaging features associated with an unfavorable postoperative seizure outcome.

A preoperative midline shift of 7 mm or more was associated with an unfavorable postoperative seizure outcome. It is most likely a surrogate parameter for significant space-occupying effects of the respective metastasis, whether it is tumor or edema related. The extent of peritumoral edema is known to be an independent predictor of postoperative seizure outcome from experience in other diseases [24]. Identification of the causative lesion can be challenging in patients with multiple brain metastases [15]. Most often, in the case of patients with BM, the indication for surgical resection is not based solely on epilepsy surgical premise, but on primarily oncological rationale (securing histology, reducing tumor burden, relieving neurologic symptoms). Nevertheless, the pathophysiologic considerations of TRE suggest that patients with multiple BMs are also more likely to have TRE, and thus the chances of success after surgical resection of one of multiple BMs are most likely to be difficult to predict [6]. In addition to the space-occupying component of edema and accompanying irritation of the surrounding parenchyma, a decrease in inhibitory neurotransmission in the peri-tumoral tissue (with or without edema) has also been discussed as an underlying explanation for tumor-related epileptogenicity [6]. An alternative mechanism explaining increased midline shift is the BM size as such. The relationship between tumor size and postoperative seizure outcome is well established in other tumor entities (e.g., meningiomas) [24]. Our volumetric data indicate a significantly larger volume of the metastatic lesions in patients with poor postoperative seizure outcome compared with BM patients with TRE and postoperative seizure freedom.

In addition to the BM volume, the ratio of intratumoral necrosis volume to total tumor volume was demonstrated to be significantly associated with postoperative seizure outcome in patients with BM and TRE. On the basis of the present data, we were able to establish a correlation between a necrosis/tumor volume ratio > 0.2 and an unfavorable seizure outcome. The cause of necrosis formation in BM and its mechanistic relationship to poor clinical outcome remain largely unknown [23]. A common hypothesis for the development of tumor necrosis is that the rapid growth of malignant cells outgrows the capacity of inherent blood supply, generating hypoxic conditions resulting in necrotic tissue areas [1]. Immunologic factors, such as the causal role of cells of the innate and adaptive immune systems in necrosis formation, have also been considered [1]. These explanatory attempts suggest an even more massive intratumoral remodeling process in the case of increased necrosis formation in BM. The associated metabolic products, as well as immunological processes, could provide the explanation for the fact that now, for the first time, a correlation between the necrosis-tumor ratio and the postoperative seizure outcome in BM patients has been successfully established in the present study.

## Limitations

The present study has several limitations. As with all retrospective studies, limitations of our study are inherent in the design and include retrospective data collection. Furthermore, there is only a small number of patients with postoperative unfavorable seizure outcome in our cohort. To reduce heterogeneity, the study cohort was appropriately narrowly defined, resulting in a very small patient sample size given the low incidence of TRE in patients with BM as well as its excellent response to surgical resection. The small sample size of the present study may severely limit the robustness of the results of the statistical analysis. Due to the inapplicability of the “one-in-ten” rule of thumb, no meaningful/conclusive multifactorial logistic regression analysis was possible. In addition, follow-up time of postoperative seizure assessment was only 3 months. Therefore, an additional shortcoming due to the retrospective nature of our data unfortunately did not allow conclusions to be drawn regarding long-term follow-up as defined by the ILAE classification system after brain surgery. Because of the oncological focus, preoperative EEG results and/or detailed epileptological evaluations are absent in the majority of patients. Furthermore, we cannot rule out that many of the patients were preoperatively treated with steroids, which could be a confounding factor in this study that affects peritumoral edema volume and subsequently the volumetric analysis. Nevertheless, the data of the present study strengthen the desire to foster future efforts to analyze larger cohorts in a multicenter design and/or by means of registry studies.

## Conclusions

Neurosurgical resection of BM is highly effective in the treatment of tumor-associated epilepsy. Additionally, the present study shows that preoperative higher tumor volumes, a midline shift > 7 mm and a necrosis/tumor volume ratio > 0.2 are associated with postoperative unfavorable seizure outcome in patients with BM and TRE. These variables might enable to preoperatively identify the subset of BM patients that are at high risk of postoperative unfavorable seizure outcome and might therefore benefit from accompanying neuro-oncological expertise during further systemical treatment regimes.

Further longitudinal studies with larger patient cohorts and in multi-center design are needed to confirm our results and asses how a neuro-oncological binding might improve seizure control and subsequently QoL in patients with BM and TRE.

## Supplementary Information

Below is the link to the electronic supplementary material.

Supplementary file1 (DOCX 26 KB)

## Data Availability

All data generated or analyzed during this study are included in this published article.
