# Supplementary material for: Tumor-associated epilepsy in patients with brain metastases: necrosis-to-tumor ratio forecasts postoperative seizure freedom
Source: Neurosurg Rev. 2021 May 14;45(1):545–51. doi: 10.1007/s10143-021-01560-y (PMC8827395; doi:10.1007/s10143-021-01560-y)
Supplement: Supplementary file 1 — Supplementary file1 (DOCX 26 KB) [file 10143_2021_1560_MOESM1_ESM.docx]

**Supplementary Table S1:** Detailed patient characteristics

| **no.** | **age/sex** | **tumor localization^1^** | **histology** | **multiple BM** | **seizure type** | **AED** | **NTR** | **EOR** | **seizure outcome** |
| --- | --- | --- | --- | --- | --- | --- | --- | --- | --- |
| 1 | f, 65 | temporal, l | bronchial carcinoma | no | simple partial | levetiracetam | ≤ 0.2 | GTR | favorable |
| 2 | f, 37 | parietal, r | carcinoma of the breast | no | simple partial | levetiracetam | ≤ 0.2 | GTR | favorable |
| 3 | m, 76 | frontal, l | bronchial carcinoma | no | simple partial | levetiracetam | ≤ 0.2 | GTR | favorable |
| 4 | f, 60 | temporal, l | small cell carcinoma of the kidney | no | simple partial | levetiracetam | ≤ 0.2 | GTR | favorable |
| 5 | f, 38 | frontal, r | carcinoma of the breast | no | simple partial | levetiracetam | ≤ 0.2 | GTR | favorable |
| 6 | f, 45 | frontal, l | carcinoma of the breast | no | simple partial | levetiracetam | ≤ 0.2 | GTR | favorable |
| 7 | f, 51 | parietal, l | carcinoma of the breast | yes | simple partial | levetiracetam | ≤ 0.2 | GTR | favorable |
| 8 | f, 55 | frontal, l | ovarian carcinoma | no | simple partial | levetiracetam | ≤ 0.2 | GTR | favorable |
| 9 | m, 63 | parietal, l | bronchial carcinoma | no | simple partial | levetiracetam | ≤ 0.2 | GTR | favorable |
| 10 | m, 61 | frontal, r | bronchial carcinoma | no | simple partial | levetiracetam | > 0.2 | GTR | unfavorable |
| 11 | m, 66 | frontal, r | colorectal cancer | no | simple partial | levetiracetam | ≤ 0.2 | GTR | favorable |
| 12 | m, 67 | parietal, l | colorectal carcinoma | no | simple partial | levetiracetam | ≤ 0.2 | GTR | favorable |
| 13 | m, 71 | insular, l | bronchial carcinoma | yes | complex partial | levetiracetam | ≤ 0.2 | GTR | favorable |
| 14 | f, 48 | temporal, l | bronchial carcinoma | no | complex partial | levetiracetam | > 0.2 | GTR | favorable |
| 15 | f, 78 | parietal, r | bronchial carcinoma | no | complex partial | levetiracetam | > 0.2 | GTR | favorable |
| 16 | f, 76 | frontal, r | colorectal cancer | no | complex partial | levetiracetam | ≤ 0.2 | GTR | favorable |
| 17 | f, 54 | occipital, l | ovarian carcinoma | no | complex partial | levetiracetam | ≤ 0.2 | GTR | favorable |
| 18 | f, 53 | frontal, r | carcinoma of the breast | no | complex partial | levetiracetam | ≤ 0.2 | GTR | favorable |
| 19 | m, 69 | frontal, l | others | yes | complex partial | valproic acid | ≤ 0.2 | GTR | favorable |
| 20 | m, 53 | parietal, l | bronchial carcinoma | no | complex partial | levetiracetam | ≤ 0.2 | GTR | favorable |
| 21 | m, 60 | temporal, r | gastric cancer | yes | complex partial | levetiracetam | ≤ 0.2 | ATL | favorable |
| 22 | m, 73 | occipital, r | prostate carcinoma | yes | complex partial | levetiracetam | ≤ 0.2 | GTR | favorable |
| 23 | m, 77 | occipital, r | bronchial carcinoma | yes | generalized | levetiracetam | ≤ 0.2 | GTR | favorable |
| 24 | m, 48 | frontal, r | melanoma | no | generalized | levetiracetam | ≤ 0.2 | GTR | favorable |
| 25 | f, 68 | frontal, r | melanoma | no | generalized | levetiracetam | ≤ 0.2 | GTR | favorable |
| 26 | f, 35 | frontal, l | thymic carcinoma | yes | generalized | levetiracetam | ≤ 0.2 | GTR | unfavorable |
| 27 | m, 66 | frontal, l | bronchial carcinoma | no | generalized | levetiracetam | ≤ 0.2 | GTR | favorable |
| 28 | m, 55 | frontal, l | bronchial carcinoma | no | generalized | levetiracetam | ≤ 0.2 | GTR | favorable |
| 29 | f, 70 | parietal, l | colorectal cancer | no | generalized | levetiracetam | ≤ 0.2 | GTR | favorable |
| 30 | m, 61 | temporal, l | bronchial carcinoma | no | generalized | levetiracetam | ≤ 0.2 | GTR | favorable |
| 31 | f, 61 | frontal, r | bronchial carcinoma | no | generalized | levetiracetam | ≤ 0.2 | GTR | favorable |
| 32 | f, 56 | frontal, l | melanoma | no | generalized | levetiracetam | ≤ 0.2 | GTR | favorable |
| 33 | f, 45 | parietal, l | melanoma | no | generalized | levetiracetam | ≤ 0.2 | GTR | favorable |
| 34 | m, 67 | frontal, r | bronchial carcinoma | no | generalized | levetiracetam | ≤ 0.2 | GTR | favorable |
| 35 | f, 64 | frontal, l | bronchial carcinoma | no | generalized | levetiracetam | ≤ 0.2 | GTR | unfavorable |
| 36 | m, 55 | temporal, l | bronchial carcinoma | yes | generalized | levetiracetam | > 0.2 | ATL | unfavorable |
| 37 | f, 75 | parietal, l | endometrial cancer | no | generalized | levetiracetam | ≤ 0.2 | GTR | favorable |
| 38 | f, 52 | temporal, r | anal cancer | no | generalized | levetiracetam | ≤ 0.2 | GTR | favorable |

AED, Antiepileptic drug; ATL, anterior temporal lobectomy in terms of a supramarginal resection; BM, Brain metastasis; EOR, extent of resection; f, female; GTR, gross-total resection; l, left; m, male; NTR, necrosis/tumor-ratio; r, right

^1^ in case of multiple metastatic lesions, tumor localization refers to the resected tumor
